# Measuring the outcome of cataract surgery: the importance of the patient perspective

**Published:** 2015

**Authors:** Robert Lindfield

**Affiliations:** Clinical Lecturer: London School of Hygiene and Tropical Medicine; Consultant in Public Health: Public Health England, UK. Robert.Lindfield@lshtm.ac.uk

**Figure:**
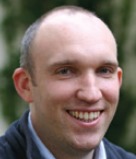
Robert Lindfield

Most eye care staff have had the pleasure of removing the pad from a patient's eye after cataract surgery and seeing their joy at having their sight restored. However, when the outcome of cataract surgery is discussed prior to surgery, the first thing most people think about is visual acuity or complications. Whilst these are critically important, they are only part of the story.

Imagine the following scenario. An 85-year-old woman presents with a visual acuity of ‘hand movements’ and dense white cataract in both eyes. She is advised to have cataract surgery. Cataract surgery in the first eye goes well with excellent technical success (a perfect capsulorrhexis, good centration of the intraocular lens, etc.) and her visual acuity improves to 1/60 in her operated eye.

Is this a good outcome? From a technical point of view it is – the surgery went well. However, from a visual acuity perspective, it is not ideal as the woman continues to have poor vision in the operated eye. What we don't know, is what the woman thought about the outcome. Was she happy? If not, why not?

## What do patients think?

We can, of course, ask patients about whether they are happy with the outcome of surgery, but we have to remember that – as humans – we are influenced by a variety of different things when considering whether we're happy with any outcome. For example, if the surgeon had told the patient that she would have perfect vision restored by surgery, would she be happy? If she had spent her life savings on surgery, would she be happy?

Understanding the patient's perspective on the visual outcome of cataract surgery can improve our cataract surgical service. It allows the hospital team to identify where improvement is required. For example, if the patient reported that the surgeon told her to expect perfect vision, then the information routinely provided by the surgeon could be reviewed and expectations better managed.

**NOTE: Remember to manage the patient's expectations. What you say will depend upon any risk factors and the presence of any co-pathology that might affect the outcome.**

**Figure:**
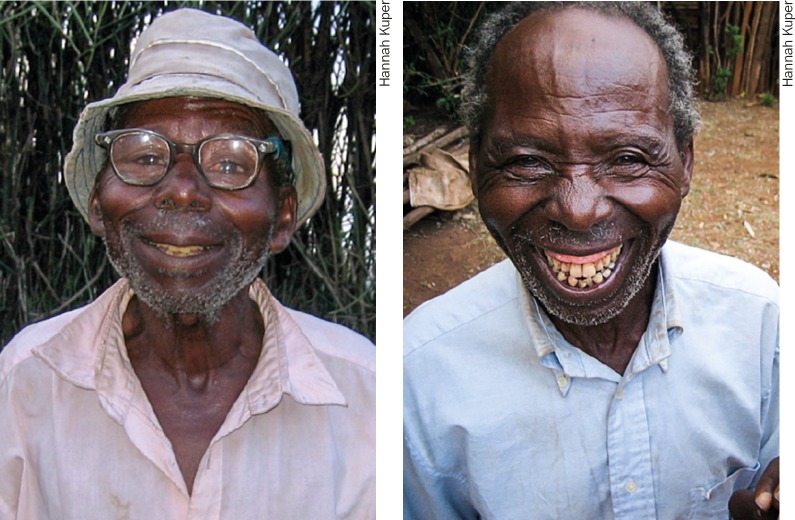
A patient before (left) and after cataract surgery. KENYA

So, how can we collect the patient's perspective on outcome? There are several ways:

1. **Comments boxes.** Many hospitals have comments boxes: patients are encouraged to write down their comments and put them in a box. The advantage of this system is that it is anonymous, so patients can be honest about their care; however, they are of limited use in countries where literacy levels are low. They also rely on ready access to paper and pen, and are less likely to be used by older patients.
2. **A questionnaire.** Questionnaires are available that capture patients' perspective on the outcome of their care. They either can be given to patients to complete (if they are able), or administered by a member of staff or volunteer. Questionnaires must be culturally appropriate and in the correct language. They rely on either the patient or carer being able to read, or one of the staff helping the patient to complete the questionnaire (which can be problematic as patients might be reluctant to raise concerns or offer criticism in the presence of a staff member).
3. **Patient interviews/exit interviews.** This involves talking with patients about their experiences at the hospital and recording their responses. Ideally, volunteers (or anyone who is **not** associated with the clinical care patients receive) should ask the questions, in order to ensure that patients feel it is safe to be honest.

## What questions to ask

The purpose of getting the patients' perspective is to find out whether he or she is satisfied with our cataract service (and will recommend it to others), and to find out how we can do better.

A simple yes/no answer (e.g.: ‘Yes, I am satisfied’, or ‘No, I am not satisfied’) is not enough. For example, patients might not have been satisfied because the bed was uncomfortable or because they were expecting their visual acuity to be perfect; these are two very different things requiring different remedial actions. In addition, satisfaction levels may be artificially high as patients might not want to be critical about aspects of their care.

It is usually more helpful to understand patients' **experience** of the cataract service. Patient experience questionnaires use quantifiable, objective measures of outcome and patient care in order to explore patients' views. A patient experience questionnaire asks a series of questions designed to try and understand the whole picture. For example, questions about:

- Information and education provided
- physical comfort
- emotional support
- respect for the patient (e.g. ‘Did the doctors/nurses sometimes talk as if you weren't there?’)
- involvement of family and friends
- continuity and transition (e.g. ‘Were you shown how to instil eyedrops before you left the hospital?’).

It is possible to find free examples of patient experience questionnaires online.^1^ These may provide a useful starting point.

## Demonstrating impact

If we want to show that surgery has changed someone's life, then just showing that their vision has improved is not enough. We need to show that they can do things that they could not do before surgery, or that they feel better.

To do this, we can do a ‘quality of life’ audit. This involves using a specially designed questionnaire and asking a randomly selected group of patients (e.g. every fifth patient) to complete it (with or without help) both before and after surgery. This makes it possible to identify any changes that have occurred and to determine the impact that surgery is having on the lives of patients.

Quality of life questionnaires have been validated (proven) to measure change in a number of areas, including people's ability to function. They ask questions such as: ‘Can you read a newspaper?’ or: ‘Can you recognise faces?’.

Quality of life questionnaires are an objective and independent method of measuring the patient's perspective on outcome. The advantage of using quality of life questionnaires is that, because we are asking for descriptions of what people can and cannot do – rather than how they feel about the outcome – there is less chance that the patient's response will be affected if the interviewer is a staff member.

Many different studies have shown that cataract surgery can improve function, and there are several questionnaires that can be used to assess this. Care has to be taken when using the questionnaires as they are context-specific. This means that each questionnaire has been developed based on the culture of the people that are being questioned. A good example is activities of daily living. In the UK, most people have a television and questionnaires often include a question on the patient's ability to watch programmes before and after surgery. Obviously this is a pointless question in places where there are few televisions. There are also difficulties in translating the questions as many languages use different types of words to describe the same thing. Therefore, care must be taken in choosing a questionnaire that is right for your country, culture and language.

At the hospital we can use quality of life questionnaires to show our patients, our staff and our supporters (including donors) that, not only do most patients see better after surgery, but most have an improved quality of life too.

## In summary

- The outcome of cataract surgery is not just about visual acuity or complications. One of the most important areas, which is rarely investigated, is the patient's perspective.
- It is important to remember that the patient's perspective is influenced by lots of different things; not just whether or not they can see.
- Quality of life questionnaires that have been designed to measure how people's functioning changes following cataract surgery are available – contact the author for details.
- Getting feedback from patients about outcome is important; however, it is only useful if it is acted on and the changes monitored to see if they have brought about the desired results. The critical outcomes of seeking patients' perspectives on their treatment, therefore, are the changes you make to your service in response to their comments.
